# Essential Amino Acid Supplement Lowers Intrahepatic Lipid despite Excess Alcohol Consumption

**DOI:** 10.3390/nu12010254

**Published:** 2020-01-19

**Authors:** Melynda S. Coker, Kaylee R. Ladd, Jimin Kim, Carl J. Murphy, Ryan DeCort, Bradley R. Newcomer, Robert R. Wolfe, Robert H. Coker

**Affiliations:** 1 Department of Natural Resources and Environment, University of Alaska Fairbanks, 505 South Chandalar Drive, Fairbanks, AK 99775, USA; mscoker@alaska.edu; 2 Department of Biology and Wildlife, University of Alaska Fairbanks, Fairbanks, AK 99775, USA; krladd@alaska.edu (K.R.L.); jkim96@alaska.edu (J.K.); 3 Institute of Arctic Biology, Department of Chemistry & Biochemistry, University of Alaska Fairbanks1930 Yukon Dr. Room 136, Fairbanks, AK 99775, USA; cjmurphy4@alaska.edu; 4 Bassett Army Community Hospital, 4076 Neely Road, FortWainwright, United States Army, Fairbanks, AK 99703, USA; rdecort@gmail.com; 5 Honors College, 1501 251Warren Service Drive, Room 105, James Madison University, Harrisonburg, VA 22807, USA; newcombr@jmu.edu; 6 Department of Geriatrics, Center for Translational Research in Aging & Longevity, Donald W. Reynolds Institute on Aging, 4301 West Markham, University of Arkansas for Medical Sciences, Little Rock, AR 72205, USA; rwolfe2@uams.edu

**Keywords:** amino acids, liver, alcohol

## Abstract

Excess alcohol consumption is a top risk factor for death and disability. Fatty liver will likely develop and the risk of liver disease increases. We have previously demonstrated that an essential amino acid supplement (EAAS) improved protein synthesis and reduced intrahepatic lipid in the elderly. The purpose of this exploratory pilot study was to initiate the evaluation of EAAS on intrahepatic lipid (IHL), body composition, and blood lipids in individuals with mild to moderate alcohol use disorder (AUD). Following consent, determination of eligibility, and medical screening, 25 participants (18 males at 38 ± 15 years/age and 7 females at 34 ± 18 years/age) were enrolled and randomly assigned to one of two dosages: a low dose (LD: 8 g of EAAS twice/day (BID)) or high dose (HD: 13 g of EAAS BID). Five of the twenty-five enrolled participants dropped out of the intervention. Both groups consumed the supplement BID for 4 weeks. Pre- and post-EAAS administration, IHL was determined using magnetic resonance imaging/spectroscopy, body composition was analyzed using dual-energy X-ray absorptiometry, and blood parameters were measured by LabCorp. T-tests were used for statistical analysis and considered significant at *p* < 0.05. While there was no significant change in IHL in the LD group, there was a significant 23% reduction in IHL in the HD group (*p* = 0.02). Fat mass, lean tissue mass, bone mineral content, and blood lipids were not altered. Post-EAAS phosphatidylethanol was elevated and remained unchanged in LD at 407 ± 141 ng/mL and HD at 429 ± 196 ng/mL, indicating chronic and excess alcohol consumption. The HD of the proprietary EAAS formulation consumed BID seemed to lower IHL in individuals with mild to moderate AUD. We suggest that further studies in a larger cohort be conducted to more completely address this important area of investigation.

## 1. Introduction

Alcohol use disorder (AUD) is a leading risk factor for death and disability and is responsible for 69 million disability-adjusted life years (DALYs) [1]. Chronic alcohol use induces hepatic steatosis in 90%–95% of individuals; liver pathology advances to cirrhosis in approximately 8%–20% of individuals with AUD and represents one of the most important clinical problems associated with AUD [2]. Ameliorating the metabolic consequences of AUD requires more than abstinence and good overall nutrition, as the majority of individuals with AUD continue to drink alcohol, increasing their risk for liver disease [3]. This clinical scenario is not dissimilar from other diseases involving lipotoxicity, in which unhealthy human behaviors require a combination of pharmaceutical, surgical, or nutritional approaches [4].

Many individuals with AUD are malnourished, and the degree of alcoholic liver disease severity correlates with the degree of malnutrition [5]. The dietary intake of protein and micronutrients often fails to meet recommended levels, even during professional, supervised recovery from AUD [6,7]. In these circumstances, a nutritional supplement designed to address the specific metabolic issues associated with the condition may provide unique benefits. A variety of nutritional supplements targeting some aspect of the AUD responses are available or have been proposed [8,9,10,11,12,13,14,15,16,17,18,19,20]. However, none have corrected the disruptions in macronutrient metabolism that lead to hepatic steatosis [11].

The beneficial influence of unique essential amino acids (EAA) on the stimulation of protein synthesis [12,13,14] and reduction in hepatic steatosis in older adults without AUD has been demonstrated [13]. Ingestion of 11 g of an EAA-based formula resulted in a 50% reduction in liver fat after only four weeks of therapy [13]. Individuals receiving the EAA formula consumed a balanced diet containing at least the minimum RDA for protein [15]. Still, the EAA exerted a profound effect on liver health. The ingestion of an EAA-based formula promotes higher plasma EAA concentrations when compared to isocaloric ingestion of intact protein, corresponding with higher net protein balance [16]. The etiology of hepatic steatosis is not identical among those at risk for metabolic disease and those who present with AUD [17]. Hepatic steatosis can occur in part because of a limitation in mitochondrial function [18], and thus impaired fatty acid oxidative capacity [19]. Impaired fatty acid oxidation results in the channeling of fatty acids into triglyceride synthesis. Based on the importance of EAA in the promotion of mitochondrial protein synthesis in the liver [20], we hypothesized that a nutritional formula enriched with either 8 or 13 g of EAA (Table 1) would reduce excess intrahepatic lipid in persons with AUD, even when alcohol consumption remained unchanged. If so, the status quo for the treatment of individuals with AUD may be modified to induce improved clinical outcomes in this segment of the population.

## 2. Materials and Methods

### 2.1. Recruitment

We utilized newspaper advertisements and posted fliers and employed a secured, telephone answering service, allowing private telephone evaluations of individual eligibility. Once it was established that the individual was a potential participant via telephone screening, we scheduled an actual screening visit at the Clinical Research and Imaging Facility (CRIF), located within the Murie Building on the UAF campus [21]. The location of the CRIF is well suited for work with volunteers from the community as there are dedicated parking spaces, and there is semi-private access with little to no student, faculty, or staff traffic. These were especially important factors in minimizing any stigmas that might be associated with the participation of volunteers in the proposed study. The screening visit included the informed consent process, blood work, and health history. Based on the eligibility criteria for mild to moderate AUD, participants were either eligible to participate or referred to their primary care physician for follow up medical care. The project (Nutrient Formulation for Liver Health: 986801-17) and all related documents were approved on 15 December 2016, by the University of Alaska Fairbanks Institutional Review Board.

We chose to restrict our age range 20–60 years of age, as much older individuals could have promoted significant variability in baseline data due to metabolic changes that occur with aging [22]. When an individual was determined to be potentially eligible via telephone interview, a screening visit was scheduled to perform a medical history and physical exam. All participants were required to have transportation to the site clinic for the screening, consent process, testing sessions, weekly checkups, and pickup/return of the essential amino acid supplement (EAAS). A capability for understanding and providing informed consent was necessary for all participants. After the screening visit, the study physician reviewed the exams and blood sample analysis, and all participants were advised of their health and eligibility status.

### 2.2. Exclusion Criteria

Any person with a pacemaker or other implanted metal, insulin-dependent diabetes, or chronic inflammatory condition were excluded. Individuals taking any type of oral contraceptive or any medication or supplement affecting glucose metabolism were excluded. Individuals with active cancers or malignancies were ineligible, as were those taking corticosteroids by mouth, injection or trans-dermally. If the study physician concluded that any medical condition or current medication represents an unacceptable risk, those individuals were excluded.

### 2.3. Study Participants

**Figure 1:**
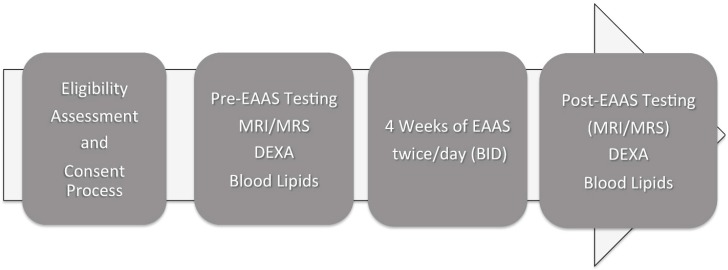
Study timeline.

Once eligibility was established during a run-in assessment period, participants were randomized to a low dose (LD) (8 g of EAAS (twice/day (BID)) or high dose (HD) (13 g of EAAS BID) supplementation and asked to undergo two testing sessions in conjunction with the 4-week supplementation phase. In each of the testing sessions (i.e., pre-supplementation and post-supplementation), participants underwent magnetic resonance imaging (MRI)/magnetic resonance spectroscopy (MRS) scans, and dual-energy X-ray absorptiometry (DXA) scans in the CRIF, and blood sampling at LabCorp (Figure 1). During the 4-week supplementation period, participants were requested to visit the CRIF at weekly intervals to retrieve their EAAS, evaluate their compliance with the protocol, and measure their weight. Compliance with the paradigm for EAAS supplementation was performed by the measurement of the weight difference in the EAAS product provided and returned each week.

### 2.4. EAAS Formulation

The proprietary EAAS formulation was produced commercially by the Prinova Group, LLC (Carol Stream, IL, USA) (Table 1). Their regulatory certifications include BRC Global Standards for Food Safety, U.S. Food and Drug Administration Regulated Facility, Kosher Supervision, Non-GMO product, Sedex Approved Supplier, The Islamic Food and Nutrition Council of America, and Safe Feed/Safe Food Certified. In addition to the EAAs, the formula also included glutamine, carnitine, niacinamide, and ascorbic acid that may be deficient in individuals with alcohol use disorder (AUD) (Table 1).

### 2.5. Intrahepatic Lipid (IHL)

**Figure 2:**
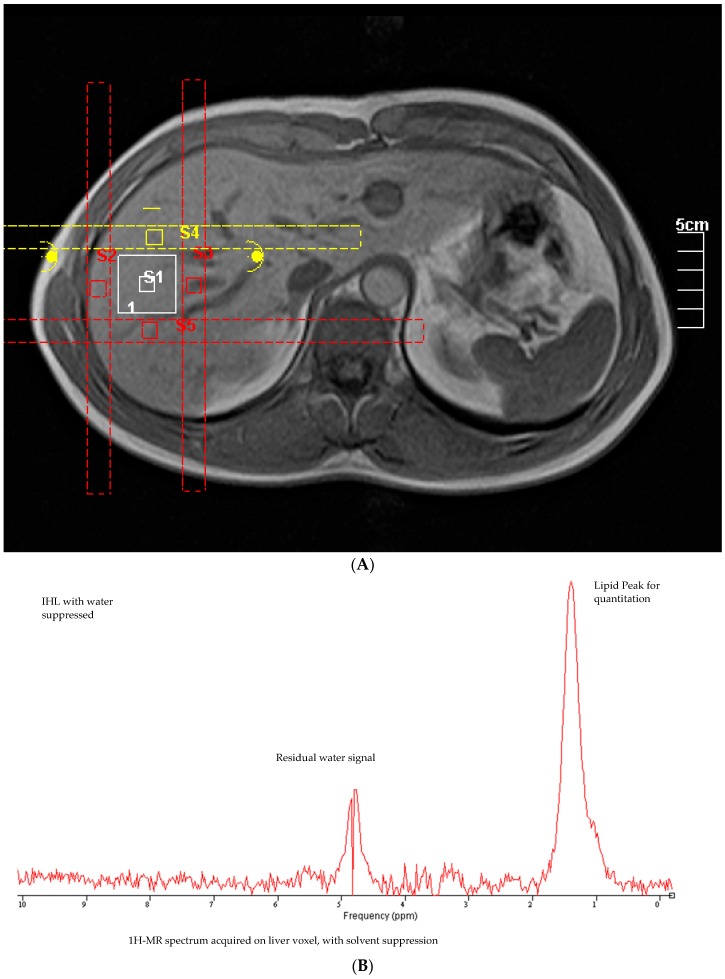
(**A**) MRI of liver detailing the location of voxel and (**B**) an example of 1-H spectroscopy derived measurement of intrahepatic lipid.

We utilized the Toshiba 1.5T Excelart/Vantage with a 1.4 m magnet and a 65.6 aperture, and IHL measurements were performed in the middle right lobe [23] (Figure 2). The scans were localized to the same area of the liver using the anatomical orientation of the hepatic blood flow and ribs, so that approximately the same area of the liver was scanned during each testing session. After a T1 scan for anatomical structures, a voxel (~30 × 30 × 30 mm) was chosen at a location free from large vessels. An optimized spectroscopy sequence was run 256 times without respiratory gating. These spectra provided an average lipid concentration measurement over the mid-right lobe. Spectra were manually phased, and the final analysis was then performed with jMRUI (Figure 2).

### 2.6. Body Weight and Composition

Total body mass was measured using an electronic scale (Health-o-Meter, St. McCook, IL, USA). A General Electric Lunar iDXA was used to determine fat mass, lean tissue mass, and bone mineral content [1].

### 2.7. Blood Measurements

Blood sampling and analysis were performed by LabCorp (1626, 30th Avenue, Fairbanks, AK, USA). LabCorp is staffed with licensed healthcare professionals, accredited by the College of American Pathologists, and licensed through the Clinical Laboratory Improvement Amendment (CLIA). In this study, serum lipid, liver, and metabolic panel analysis were included. The lipid panel included total cholesterol, high-density lipoprotein cholesterol (HDL), low-density lipoprotein cholesterol (LDL), very low-density lipoprotein cholesterol (VLDL), and triglycerides. The liver panel consisted of albumin, alanine transaminase (ALT), aspartate transaminase (AST), bilirubin (total and direct), and total protein. The metabolic panel included blood urea nitrogen (BUN), calcium, carbon dioxide, chloride, creatinine, glucose, potassium, and sodium. Whole blood phosphatidylethanol (Peth) was measured to ascertain the level of alcohol consumption.

### 2.8. Statistical Analysis

Data were analyzed using Microsoft Excel, General Electric iDXA Encore, and Prism 5 software. Data are presented as means ± SD. Two sample homoscedastic t-tests were used to evaluate potential differences between groups. In order to generate statistical data for this exploratory pilot study, paired t-tests were utilized to compare differences in pre-supplementation and post-supplementation within groups.

## 3. Results

Research Participants. We enrolled 25 research participants (18 males and 7 females) with mild to moderate AUD for this study. Seventeen individuals completed all aspects of the study; five dropped out and three participants failed to get their post-supplementation blood sampling. Based on EAAS weigh back data, the average daily compliance to EAAS was 85% ± 15% and 83% ± 8% in LD and HD groups, respectively. The average weight, body mass index, and body composition was similar between groups and did not change with EAAS.

**Figure 3:**
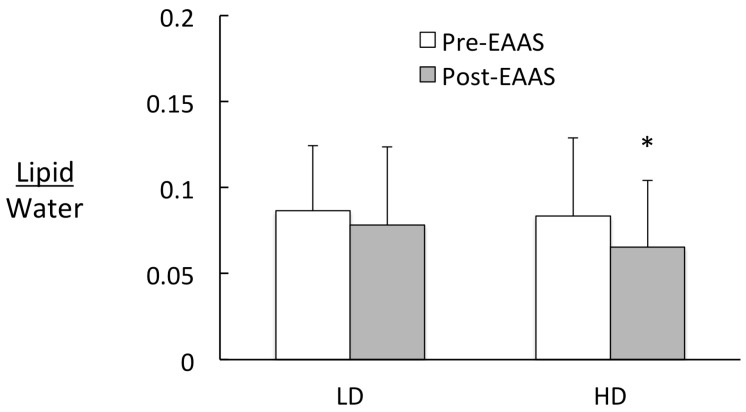
Intrahepatic lipid in low dose (LD) and high dose (HD) pre- and post-essential amino acid supplement (EAAS). * Represents a significant difference between pre- and post-EAAS (*p* = 0.02).

Intrahepatic Lipid. IHL was elevated at baseline in both groups and decreased by 23% in the HD group with EAAS, presumably due to increased protein synthesis [24] (Figure 3). The significant reduction in IHL represented approximately half of the reduction needed to return IHL to normal levels [25,26] but our observation is limited by the relatively small sample and the lack of a control group.

Blood parameters. Total cholesterol, LDL-cholesterol, VLDL-cholesterol, HDL-cholesterol, and triglycerides were all within normal limits and did not differ between groups and did not change with EAAS (Table 2). Except for blood Peth, all other blood parameters were also within normal limits and were not altered by EAAS, indicating the lack of any undesirable effects of EAAS on lipid, liver or metabolic function (Table 2). It is indeed possible that variations in some of these parameters may have precluded an opportunity to reject the null hypothesis and this warrants further examination of baseline dietary intake and/or physical activity levels. Blood Peth levels were elevated but not different between groups and were not altered from pre- to post-supplementation in either group. Blood Peth levels above 400 ng/dl confirmed alcohol misuse in all participants (Table 2) [27].

## 4. Discussion

The primary focus of this exploratory pilot investigation was to determine whether EAAS BID would decrease IHL in individuals with mild to moderate AUD. We have now demonstrated that 13 g of EAAS provided BID significantly reduced IHL without manipulation of dietary intake, change in habitual alcohol consumption, or any form of behavioral modification. On the other hand, 8 g of EAAS BID did not influence IHL. Given the overall normal ranges for blood parameters except for Peth, it was not surprising that there were no changes in circulating lipid, liver, or metabolic blood parameters in either group. Future studies in a larger cohort with a longer intervention paradigm are now warranted to evaluate whether these efficacious alterations persist and whether lower doses of EAAS could IHL.

Several studies have posited the beneficial influence of essential amino acids on the mitigation of hepatic steatosis [28,29,30,31]. To date, this work has focused on nonalcoholic hepatic steatosis, which also describes an excessive accumulation of IHL similar to alcoholic hepatic steatosis [32]. Recommendations suggest that a diet containing foods with more favorable glycemic indexes and energy values coupled with reductions in saturated fat intake should be combined with increased exercise and weight reduction to lower IHL in those with non-alcoholic hepatic steatosis [33]. Unfortunately, adherence to behavioral modification and/or lifestyle intervention has proven extremely difficult [34] for many individuals with hepatic steatosis; regardless of the underlying pathology.

The mechanisms responsible for EAAS-mediated improvements in IHL in the current study may be linked to their influence on mitochondrial biogenesis [35] and/or the complex modulation of AMPKα, mTOR, sirtuin-1, and PPAR-γ, all of which regulate pathways of fatty acid kinetics [31]. The provision of EAAS has also been demonstrated to reduce insulin resistance, a common factor implicated in the excessive deposition of lipids in the liver [36]. In the current study, subjects had normal blood glucose concentrations and thus mitigation of insulin resistance was unlikely to have been a significant factor in reducing IHL. Whereas a nonspecific increase in overall dietary protein intake might introduce unnecessary nonessential amino acids linked to excess ammonia and urea production [37], provision of the higher dose of EAAS BID may improve IHL through augmentation of mitochondrial volume and turnover [38].

Suppression of mTORC1 via alcohol intake presents a completely different physiological circumstance [39] than the association between BCAAs and increased mTORC1 in obesity [40]. Given that mTORC1 is vitally important in the stimulation of mitochondrial biogenesis and the corresponding increment in oxidative metabolism needed to support anabolism of metabolic machinery [41], it is not surprising that the provision of EAAS provided beneficial alterations in IHL. While the risks for the development of metabolic syndrome in individuals with AUD are two-fold higher than the rest of the population [42], this is likely due to interactions between dietary intake, lack of physical activity, and alcohol intake [43]. The serum lipids in our participants were within normal limits or borderline high. In the absence of weight loss, it was relatively unlikely for cholesterol or triglycerides to decrease in non-obese individuals.

Regardless of the lack of therapeutic benefit of EAAS on serum lipids that were not elevated by established clinical standards, the beneficial reduction in IHL was significant. Other studies have demonstrated similar benefits in individuals at risk for metabolic diseases [31,44], but this is the first study to our knowledge that has established a link between EAAS and the reduction of IHL in individuals with AUD. It is our assertion that EAAS may have likely improved cytosolic concentrations of amino acids in the liver, which positively altered mitochondrial protein synthesis as previously demonstrated in pre-clinical studies [20]. Combined with the impact of EAAS on transcription via their influence on mTOR [45], these molecular avenues may have allowed EAAS to exert its beneficial influence on mitochondrial biogenesis. While these possibilities are intriguing, our study did not specifically evaluate EAAS-induced alterations in mitochondrial function but rather provide an impetus for future studies that could define the precise mechanisms.

We recognize that the lack of strict dietary control and/or management of physical activity patterns represent the limitations of our study design as both factors may affect IHL [46,47]. Nonetheless, the BMI of our participants indicated that obesity was unlikely to be a contributing factor in the accumulation of excess IHL and the exemption of individuals with diabetes eliminated the influence of that particular disease process on liver metabolism. Instead, we chose to focus on the efficacy of a simple nutritional therapy (i.e., EAAS) on IHL in the context of alcohol misuse (as supported by elevated and stable Peth levels) [48]. This strategy was consistent with our intention to minimize the complexity of the intervention, maximize EAAS compliance, and improve the potential for practical applications based on solid clinical evidence.

Finally, why provide a supplement that could somewhat offset the deleterious influence of alcohol on liver metabolism? The answer to this important question lies in the fact that less than 7% of individuals with AUD will actually seek professional treatment [49], even though early mitigation of steatosis may delay the progression of alcoholic liver disease [50]. Despite the modest number of individuals who actually seek treatment, progress in early treatment has been made, including reduced stigma for behavioral services, classification of excess alcohol use as a disorder, as well as providing confidential access to national substance abuse and distress helplines. Educational initiatives have also been implemented to improve the recognition of excess alcohol use [2]. As indicated by aminotransferase levels within normal limits in our own participants, hepatic steatosis will likely exist prior to the ability to detect liver damage via blood sampling/evaluation [51,52]. Given that treatment for this disorder is complex [53], therapeutic options should use a multifaceted approach to decrease the pernicious influence of alcohol-induced hepatic steatosis on health outcomes. Otherwise, the complex etiology of alcohol-related liver disease; including poor nutritional status [54], impairments in fatty acid oxidation [52], and perturbations in the NADH: NAD+ ratio will continue to worsen the condition of the liver [55].

## 5. Conclusions

Our study has some important limitations that include small sample size and the lack of baseline dietary information. Despite these factors, we have demonstrated that EAAS reduces IHL in individuals with AUD despite continual and consistent alcohol consumption. Future clinical studies should be directed toward a larger cohort with variable levels of dyslipidemia and longer EAAS interventions.

## Figures and Tables

**Table 1:** Essential amino acid supplement (EAAS) formulation.

	Low Dose (8 g)	High Dose (13 g)
*Essential Amino Acids* (mg)		
Leucine	1483	2410
Isoleucine	732	1190
Valine	954	1550
Methionine	345	560
Histidine	363	590
Lysine	1102	1790
Threonine	548	890
Phenylalanine	911	1480
Tryptophan	148	240
*Other Ingredients* (mg)		
Glutamine	123	200
Carnitine	308	500
Niacinamide	77	125
Ascorbic Acid	308	500
Caffeine	62	100
Sucralose	QS	QS
Acesulfame Potassium	QS	QS
Citric Acid	QS	QS
Malic Acid	QS	QS
Sodium Citrate	QS	QS
Flavors	QS	QS

**Table 2:** Clinical characteristics.

	Pre-EAAS LD	Post-EAAS LD	Pre-EAAS HD	Post-EAAS HD	NORMAL RANGE
Sex (F/M)	4/4	4/4	2/7	2/7	-
Weight (kg)	81 ± 11	81 ± 10	74 ± 11	74 ± 12	-
Body Mass Index (kg/m^2^)	26 ± 3	26 ± 1	25 ± 4	25 ± 4	18.5–25.9
Fat Mass (kg)	20 ± 7	19 ± 7	19 ± 7	19 ± 7	-
Lean Tissue Mass (kg)	57 ± 11	58 ± 11	51 ± 8	51 ± 8	-
Total Cholesterol (mg/dL)	187 ± 26	176 ± 21	184 ± 32	186 ± 35	100–199
LDL-cholesterol (mg/dL)	100 ± 22	87 ± 21	105 ± 17	103 ± 21	0–99
VLDL-cholesterol (mg/dL)	23 ± 13	35 ± 24	22 ± 22	23 ± 20	5–40
HDL-cholesterol (mg/dL)	57 ± 13	55 ± 14	58 ± 12	60 ± 12	>39
Triglycerides (mg/dL)	152 ± 113	175 ± 115	107 ± 13	117 ± 98	0–149
Albumin (g/dL)	4.5 ± 0.4	4.4 ± 0.3	4.5 ± 0.2	4.6 ± 0.1	3.5–5.5
ALT (IU/L)	25 ± 11	18 ± 7	20 ± 10	18 ± 9	0–44
AST (IU/L)	23 ± 7	19 ± 5	22 ± 6	29 ± 21	0–40
Bilirubin-total (mg/dL)	0.5 ± 0.3	0.5 ± 0.3	0.7 ± 0.6	0.6 ± 0.2	0.0–1.2
Bilirubin-direct (mg/dL)	0.1 ± 0.1	0.1 ± 0.1	0.2 ± 0.1	0.2 ± 0.0	0.0–0.4
Protein-total (g/dL)	7.1 ± 0.4	6.7 ± 0.4	7.0 ± 0.4	6.8 ± 0.4	6.0–8.5
BUN (mg/dL)	14 ± 4	14 ± 4	16 ± 5	16 ± 4	6–24
Calcium (mg/dL)	9.4 ± 0.2	9.5 ± 0.2	9.5 ± 0.3	9.4 ± 0.3	8.7–10.2
Carbon Dioxide (mmol/L)	24 ± 1	24 ± 1	24 ± 2	25 ± 1	20–29
Chloride (mmol/L)	102 ± 1	100 ± 1	101 ± 2	101 ± 2	96–106
Creatinine (mg/dL)	0.9 ± 0.1	0.9 ± 0.1	0.9 ± 0.2	0.9 ± 0.1	0.76–1.27
Glucose (mg/dL)	90 ± 7	90 ± 5	85 ± 5	86 ± 7	65–99
Potassium (mmol/L)	4.5 ± 0.4	4.3 ± 0.2	4.3 ± 0.2	4.4 ± 0.2	3.5–5.2
Sodium (mmol/L)	141 ± 1	140 ± 2	141 ± 1	142 ± 1	134–144
Peth (ng/mL)	407 ± 141	429 ± 196	429 ± 196	422 ± 224	<20
